# Echocardiographic index E/e’ in association with cerebral white matter hyperintensity progression

**DOI:** 10.1371/journal.pone.0236473

**Published:** 2020-07-27

**Authors:** Woo-Jin Lee, Keun-Hwa Jung, Young Jin Ryu, Soon-Tae Lee, Kyung-Il Park, Kon Chu, Manho Kim, Sang Kun Lee, Jae-Kyu Roh

**Affiliations:** 1 Department of Neurology, Seoul National University Hospital, Seoul, South Korea; 2 Department of Radiology, Seoul National University Bundang Hospital, Seongnam-si, Gyeonggi-do, South Korea; 3 Department of Neurology, Seoul National University Hospital Healthcare System Gangnam Center, Seoul, South Korea; 4 Department of Neurology, The Armed Forces Capital Hospital, Seongnam-si, Gyeonggi-do, South Korea; University at Buffalo, UNITED STATES

## Abstract

Cerebral white-matter hyperintensities (WMHs) on MRI is associated with reduced compliance of the cerebral arterioles. We hypothesized that an echocardiography index for left ventricular (LV) diastolic function, E/e’, might reflect the cerebral arteriolar compliance and evaluated the association between E/e’ and long-term progression rate of the cerebral WMH volume. This retrospective study included individuals who were ≥ 50 years of age, with a preserved LV ejection fraction (≥ 50%) and neurological function status (modified Rankin scale score ≤1), and underwent initial and follow-up MRI evaluations within intervals of 34–45 months. Baseline clinical, laboratory, and echocardiography markers such as ejection fraction, LV mass index, and E/e' were obtained. WMH volume progression rate between the baseline and follow-up MRIs was designated as the outcome factor. 392 individuals (57.1% men; mean age: 66.7±8.4 years) were followed-up for 38.2±3.4 months. The mean WMH volume progression rate was 1.35±2.65 mL/year. The log-transformed value of WMH volume progression rate was linearly associated with the log-transformed E/e’ (B coefficient = 0.365; 95% confidence interval [CI] 0.180−0.551; *P* = 0.001), along with the log-transformed values of baseline WMH volume (B = 0.142; 95% CI 0.106−0.179; *P*<0.001) and glomerular filtration rate (B = -0.182; 95% CI -0.321−0.044; *P* = 0.010). Additionally, a subgroup with an E/e’ ≥15 exhibited a significantly higher WMH progression rate compared to the subgroups with lower E/e’ values (*P*<0.001), especially in the lower quartiles (quartiles 1 and 2) of the baseline WMH volume. We concluded that echocardiographic marker E/e’ is associated with the long-term progression rate of cerebral WMHs in population with preserved LV systolic function.

## Introduction

Cerebral white matter hyperintensity (WMH) on the brain MRI is a prevalent aging-related phenomenon and is implicated in various medical complications. [1–3] Major pathomechanisms underlying the WMH progression include chronic hypoperfusion in the brain parenchyma, [4] disruption of the blood-brain barrier (BBB), [5] and dysfunction of the perivascular (glymphatic) metabolite clearance system, [6] which are all pathomechanistically related with the reduced pulsation of the cerebral arterioles. [7, 8] As the long-term remodeling process in cerebral arterioles results in reduced elasticity in vessel walls and diminished pulsation, [1, 9, 10] markers that reflect arteriolar compliance have been evaluated in relation to WMH progression. [8, 11, 12] Although conventional vascular risk factors were recognized to be associated with WMH progression, those factors did not account for much of the risk. [1, 2, 13, 14] Therefore, a noninvasive marker that reflects the arteriolar remodeling process and represents arteriolar compliance might be useful in predicting and modulating the progression of WMH in a timely fashion.

Diastolic dysfunction of the left ventricle (LV) of the heart is very prevalent in the geriatric population and precedes various cardiac complications, such as congestive heart failure, coronary artery disease, atrial fibrillation, and mortality. [15–18] As both the LV diastolic dysfunction and the reduced cerebral arteriolar compliance are the consequences of long-term myocardial or vascular remodeling, [8, 19, 20] a marker that evaluates the LV diastolic function might also reflect the compliance of the cerebral arterioles and be associated with the cerebral WMH progression. Although a comprehensive echocardiographic evaluation using multiple parameters is required to adequately measure the LV diastolic function, [18, 21, 22] the ratio between early mitral inflow velocity (E) and mitral annular early diastolic velocity (e’, E/e’) has been recognized as one of the major echocargiography markers for evaluating LV diastolic function. [17]. Therefore, investigating whether the E/e’ is also associated with the progression of WMH might provide a substantial expansion of its clinical utility and further elucidate the pathophysiology of WMH.

In this study, we hypothesized that E/e’ might reflect the compliance of cerebral small arteries. In this regard, we evaluated the association between the E/e’ and the progression of cerebral WMH in a population without overt cardiovascular event.

## Materials and methods

### Study population

This retrospective cohort study initially included all consecutive individuals who visited Seoul National University Hospital between January 2010 and May 2014 for a health check-up program, were ≥ 50 years of age, underwent baseline MRI/magnetic resonance angiography (MRA) and transthoracic echocardiography within 3 months of interval, and underwent follow-up MRIs at 34–45 month intervals from the baseline MRI evaluation. Among the initially included 671 individuals, the final study population was defined according to the following criteria: (1) absence of echocardiography or symptomatic evidence of valve disease, myocardial infarction, intra-cardiac shunt disease, or pericardial disease; (2) preserved LV systolic function (ejection fraction ≥ 50%) [19]; (3) no significant (≥ 50%) stenosis in the internal carotid arteries (ICAs) or the middle cerebral arteries (MCAs) at the initial MRA; (4) able to carry out daily activities independently without a neurological deficit; (5) no history of stroke other than an old lacunar stroke (> 90 days); and (6) no chronic disorders involving the central nervous system (CNS). Patients with coronary artery disease without an established medical history of myocardial infarction were included. Based on the criteria, 73 subjects with heart disease, 51 with reduced LV systolic function, 69 with significant stenosis in the ICAs or the MCAs, 15 with fixed neurological deficit or incapable of independent daily living, and 71 patients with a territorial stroke history or a demonstrated chronic CNS disease were sequentially excluded. Finally, the remaining 392 individuals were included for the study analysis. The indications for the MRI/MRA and echocardiography evaluations are demonstrated in S1 Table. The design of this study was approved by the institutional review board of Seoul National University Hospital. The requirement for informed consent was waived as all information was anonymized and de-identified prior to our analysis.

### Acquisition of clinical data

Demography information and clinical profiles, including the presence of a stroke history, hypertension, diabetes mellitus, hyperlipidemia, subclinical coronary artery disease, smoking in the past 5 years, and the presence of atrial fibrillation were evaluated. Subclinical coronary artery disease was defined as presence of any plaque or stenosis in coronary artery according to the CT coronary angiography evaluation. [23] Regular use of antithrombotic agents, beta-blockers, or angiotensin-converting enzyme inhibitor/aldosterone-receptor blocker (ACEI/ARB) were also reviewed.

### Echocardiography evaluation

A 2-dimensional echocardiography examination was performed by skilled echocardiographers using commercially available echocardiography devices with a 2.5-MHz transducer and a standard multi-frequency probe with comprehensive LV measurements made from M-mode in the parasternal long-axis following a standardized protocol. Left ventricular mass index (LVMI) was measured as: $\frac{0.8*\{\left[1.04({LVEDd+IVSd+PWd)}^{3}-{(LVEDd}^{3})\right]\}+0.6}{body\;surface\;area}$ where LVEDd is left ventricular end-diastolic diameter (mm), IVSd is interventricular septal thickness at end-diastole (mm), and PWd is posterior wall thickness at end-diastole (mm). [17, 24] The LV systolic ejection fraction was also measured. [17, 24]

### Measurement of E/e’

E/e’ was defined as the maximum velocity of the early trans-mitral filling flow at diastole (E-wave) divided by the maximum velocity of the septal mitral annulus at early diastole (e’-wave). Pulsed spectral Doppler measurements using a 5 mm sample volume were defined at the tip of the mitral leaflets parallel to ventricular inflow during diastole, with a sweep speed of 100 mm/s. To measure the velocity of mitral annular motion, tissue Doppler data were obtained at the level of the mitral annulus over the septal walls. e’ was identified as the early negative deflection during diastole on the Doppler tracing. The mean values of four different cardiac cycles were used in the analysis to minimize the effect of beat-to-beat variation or respiration.

### Laboratory measurements

At the initial visit, the glomerular filtration rate (GFR) was measured using the Chronic Kidney Disease-Epidemiology Collaboration equation. [13] Total cholesterol levels and low-density lipoprotein (LDL) levels were obtained after overnight fasting. Blood pressure parameters including systolic blood pressure (SBP, mmHg), diastolic blood pressure (DBP, mmHg), and pulse pressure (PP, mmHg), obtained at the initial visit and after approximately 12-month intervals were averaged in the analyses.

### Magnetic resonance imaging

1.5-T MRI imaging units with an 8-channel head coil (Intera Achieva; Philips Medical Systems, Best, the Netherlands, Signa Excite; GE Medical Systems, Milwaukee, WI, USA, and Verio; Siemens Medical Solutions, Erlangen, Germany) were used. MRI protocols included axial T1/T2 weighted images, gradient-echo, fluid-attenuated inversion recovery (FLAIR), intracranial time-of-flight (TOF) angiography, as well as a neck MRA. FLAIR images were acquired using a parameters set including slice thickness of 4.0 mm, no slice gap, 24–27 slices covering the entire brain, a field-of-view (FOV) = 240 mm×240 mm repetition time/echo time (TR/TE) = 9000–9900/97–163 ms, and matrix = 220×220. TOF and neck MRAs were reviewed to evaluate the presence of a significant stenosis in the middle cerebral artery (MCA) and the internal carotid artery (ICA). Images were reviewed by a radiologist (YJR, 8 years of experience) blinded to all patient data.

### Volumetric analysis

FLAIR images of baseline and follow-up MRI evaluations were registered in an off-line workstation. Image co-registration on a brain template was performed using a semi-automatized software NeuRoi (Nottingham university, Nottingham, UK), according to the manual provided by the program manufacturer. [25, 26] WMH lesions were defined as high signal-intensities in the white matter area and old lacunar infarctions appeared as lesions with clean or sharp edges with a dark signal in the center were excluded. [9, 14] WMH lesions were outlined by a neurologist (WJL, 8 years of experience) and the total intracranial volume and the total WMH volume were measured using a semi-automatized software NeuRoi as previously described. [12, 27] During the analyses, the evaluator was blinded to whether the images were baseline or follow-up and to all clinical information. The WMH progression rate was calculated by subtracting the baseline the WMH volume from the follow-up WMH volume and dividing it by the MRI evaluation intervals (mL/year). The reproducibility of WMH volume measurement was evaluated in the previous studies, which was excellent. [12, 27]

### Statistical analysis

SPSS 23.0 (SPSS Inc., Chicago, IL, USA) was used for all statistical analyses. [28] Pearson’s or Spearman's rank correlation analysis was applied to measure the correlations between continuous variables and the WMH progression rate. For categorical variables, mean WMH progression rate and other clinical and laboratory parameters were compared among subgroups using Mann–Whitney *U* test or analysis of variance (ANOVA).

Variables with *P* values < 0.10 in univariate analyses were included in following multivariate linear regression analysis using a backward elimination method. In the multivariate analysis, dependent variables including baseline WMH volumes, E/e’, LVMIs, PPs, total cholesterol, and GFR were log transformed to obtain a normal distribution. To obtain a normal distribution of the dependent variable, log transformation of the (WMH progression rate +1 [mL/year]) was performed. After obtaining the linear regression model, standardized predicted values and standardized residuals were plotted to check the assumption for homoscedasticity. Scatterplots of the dependent variables and the significantly associated independent variables were drawn to check the assumption for linearity. To examine the assumption for the normal distribution of the errors between observed and predicted values, a normal probability (P-P) plot of the standardized residuals were drawn. To access the multicollinearity between variables, the variance inflation factor (VIF) was measured, where a value of >3.00 indicates a significant collinearity. The WMH progression rate was evaluated separately for the quartiles of baseline WMH volume and the E/e’ intervals (E/e’ < 8, 8 ≤ E/e' < 15, and E/e’ ≥ 15), [24] and compared using Analysis of Covariance (ANCOVA) analysis adjusting for age and sex. For every analysis, *P* values < 0.05 were considered statistically significant.

## Results

In 392 individuals (224 [57.1%] men; mean age: 66.7±8.4 years, range: 50 − 87 years), the baseline WMH volume was 10.01±15.97 mL and the mean E/e’ was 10.3±3.8. For baseline MRI analysis, Intera Achieva was used in 164 (41.8%), Verio in 129 (32.9%), and Signa Excite in 99 (30.1%) patients. On follow-up MRIs, performed at an interval of 38.2±3.4 months from the initial MRI, the mean WMH volume change was 4.23±8.32 mL and the WMH progression rate was 1.35±2.65 mL/year (Table 1). Clinical, laboratory, and WMH profiles were comparable among the subgroups with different MRI scanners (S2 Table).

**Table 1:** Clinical, laboratory, and white matter hyperintensity profiles of the study population.

Clinical profiles	N = 392
Age, years	66.7±8.4
Male sex	224 (57.1)
Previous lacunar stroke	111 (28.3)
Hypertension	254 (64.8)
Diabetes mellitus	134 (34.2)
Hyperlipidemia	129 (32.9)
Subclinical coronary artery disease	57 (14.5)
Smoking in past 5 years	49 (12.5)
Atrial fibrillation	46 (11.7)
Use of antithrombotic agents	299 (76.3)
Use of beta-blockers	116 (29.6)
Use of ACEI/ARB	148 (37.8)
**Laboratory findings**	
Total cholesterol, mg/dL	163.1±37.1
LDL cholesterol, mg/dL	90.5±28.6
Glomerular filtration rate, mL/min	85.6.±22.5
Systolic BP, mmHg	134.9±22.6
Diastolic BP, mmHg	78.8±10.7
Pulse pressure, mmHg	56.1±16.9
**Echocardiography profiles**	
Ejection fraction, %	62.5±6.3
E, cm/s	64.7±27.1
e’, cm/s	6.6±1.7
E/e’	10.3±3.8
Left ventricular mass index, g/m^2^	91.2±21.2
**Radiography profiles**	
Baseline WMH volume, mL	10.01±15.97
Median (IQR)	3.75 (1.22−13.56)
WMH volume change, mL	4.23±8.32
Median (IQR)	1.25 (0.02−4.70)
WMH progression rate, mL/year	1.35±2.65
Median (IQR)	0.39 (0.01−1.50)

Data are reported as number (percentage) or as mean± standard deviation. LDL: low-density lipoprotein, BP: blood pressure, ACEI/ARB: angiotensin-converting enzyme inhibitor/aldosterone-receptor blocker, WMH: white matter hyperintensity.

Correlation analyses revealed that the WMH progression rate was significantly associated with age, baseline WMH volumes, E and e’ wave velocities, E/e’ ratios, SBPs, PPs, and GFRs (Table 2). For the categorical variables, previous lacunar stroke history, presence of hypertension, diabetes mellitus, hyperlipidemia, and use of ACEI/ARB were associated with a higher WMH progression rate (Table 3). In following multivariate linear regression analysis, the log-transformed E/e’ was significantly associated with log-transformed WMH progression rates (B coefficient = 0.365; 95% CI 0.180−0.551; *P* = 0.001) along with the log-transformed values of baseline WMH volumes (B = 0.142; 95% CI 0.106−0.179; *P*<0.001) and GFR (B = -0.182; 95% CI -0.321−-0.044; *P* = 0.010, Table 4). However, age, LVMI, SBP, PP, or conventional cerebrovascular risk factors were not significantly associated with WMH progression. Scatterplots returned linear association of log-transformed WMH progression rate with log-transformed values of baseline WMH volume, E/e’ and GFR. In the scatterplot of the standardized predicted values and the standardized residuals, a random and even distribution of the standardized residuals around the zero line was observed. The P-P plot distribution of standardized residuals was near the comparison line. VIF values for each variable were <2.0.

**Table 2:** Correlation coefficients of white matter hyperintensity progression rate with continuous variables.

	Correlation coefficiency	*P*
Age (year)	0.245	<0.001**
Baseline WMH volume, mL	0.409	<0.001**
Ejection fraction, %	-0.006	0.916
E, cm/s	0.183	0.001**
e’, cm/s	-0.120	0.031*
E/e’	0.318	<0.001**
Left ventricular mass index, g/m^2^	0.096	0.087
Systolic BP, mmHg	0.164	0.003**
Diastolic BP, mmHg	0.068	0.222
Pulse pressure, mmHg	0.176	0.002**
Total cholesterol, mg/dL	-0.093	0.095
LDL cholesterol, mg/dL	-0.062	0.265
Glomerular filtration rate, mL/min	-0.155	0.005**

WMH: white matter hyperintensity, BP: blood pressure, LDL: low-density lipoprotein

*P<0.05

**P<0.01.

**Table 3:** Univariate analyses for categorical parameters associated with white-matter hyperintensity progression.

	WMH progression rate, mL/year		
	Yes	No	*P*
Male sex	0.36 (0.00−1.36)	0.39 (0.01−1.57)	0.945
Previous lacunar stroke	0.80 (0.08−2.69)	0.22 (0.00−1.20)	0.025*
Hypertension	0.56 (0.01−1.93)	0.13 (0.00−0.90)	0.002**
Diabetes mellitus	0.64 (0.02−2.87)	0.25 (0.00−1.18)	0.013*
Hyperlipidemia	0.75 (0.03−2.35)	0.23 (0.00−1.20)	0.002*
Subclinical coronary artery disease	0.61 (0.03−1.33)	0.33 (0.01−1.53)	0.632
Smoking in past 5 years	0.65 (0.00−3.00)	0.33 (0.01−1.35)	0.608
Atrial fibrillation	0.64 (0.01−2.68)	0.33 (0.01−1.35)	0.688
Use of antithrombotic agents	0.52 (0.01−1.63)	0.15 (0.00−1.01)	0.165
Use of beta-blockers	0.39 (0.01−0.97)	0.28 (0.01−0.85)	0.955
Use of ACEI/ARB	0.46 (0.02−1.22)	0.23 (0.00−0.82)	0.021*

Data are reported as median (interquartile range, IQR).

*P<0.05

**P<0.01. ACEI/ARB: angiotensin-converting enzyme inhibitor/aldosterone-receptor blocker.

**Table 4:** Linear regression analyses for factors associated with log-transformed white-matter hyperintensity progression rate.

	B (95% CI)	β	P
	-0.040 (-0.912−0.832)		0.928
Age, years	0.005 (-0.003−0.013)	0.063	0.196
Baseline WMH volume, mL^†^	0.142 (0.106−0.179)	0.370	<0.001**
E/e’^†^	0.365 (0.180−0.551)	0.184	0.001**
Glomerular filtration rate, mL/min^†^	-0.182 (-0.321−0.044)	-0.118	0.010*

R^2^ = 0.312 and *P*<0.001 for the linear regression equation.

B: unstandardized coefficient, β: standardized coefficient, CI: confidence interval, WMH: white matter hyperintensity

^†^The variables were log-transformed to obtain a normal distribution

**P<0.01.

**Fig 1:**
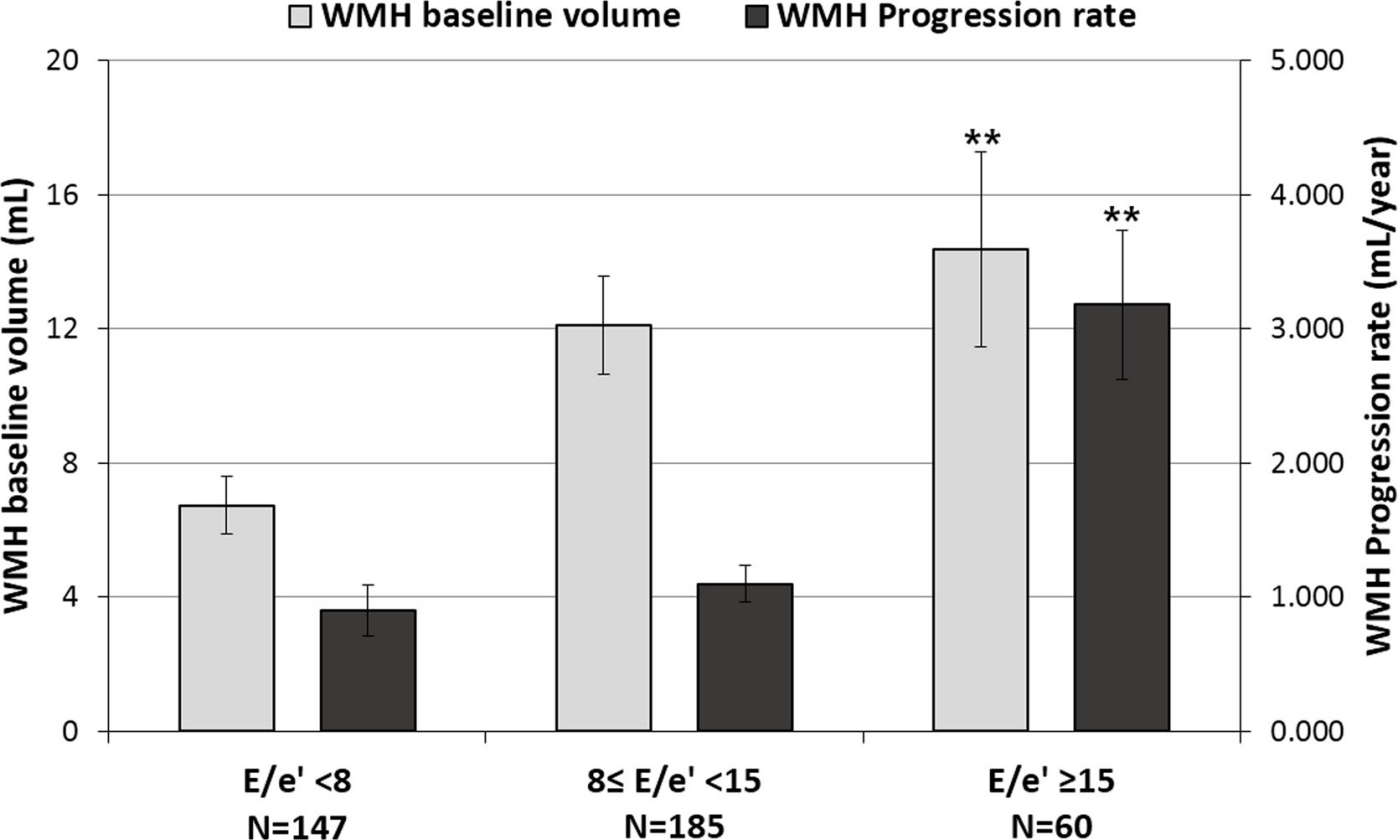
Profiles of baseline White Matter Hyperintensity (WMH) volume and WMH progression rate according to E/e’ ratio. Horizontal lines above the bars denote standard errors. **P<0.01, compared to the subgroups with low (E/e’< 8), medium (8 ≤ E/e' < 15) E/e’. WMH: white matter hyperintensity.

**Fig 2:**
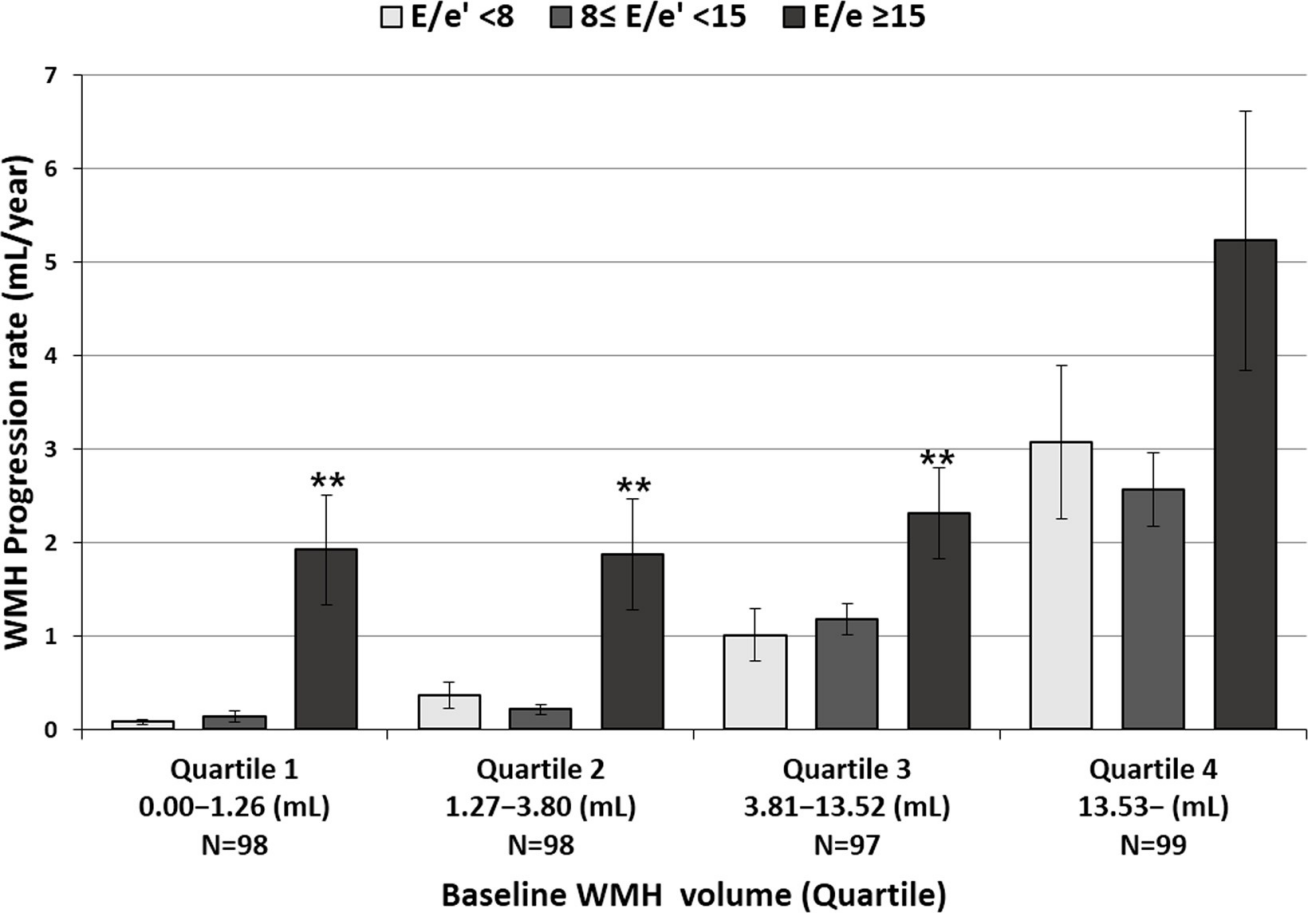
Rates WMH progression according to E/e’ ratio and quartiles of baseline WMH volume. Horizontal lines above the bars denote standard errors. **P<0.01, compared to the subgroups with low (E/e’< 8), medium (8 ≤ E/e' < 15) E/e’. WMH: white matter hyperintensity.

When the study population was grouped according to the E/e’ values into low (E/e’<8), medium (8≤ E/e'<15), and high (E/e’ ≥15), the subgroup of high E/e’ was significantly related to a higher baseline WMH volume and a faster WMH progression rate (*P* = 0.008 and *P*<0.001, respectively, Fig 1). ANOVA analysis found that the E/e’ ≥15 subgroup was associated with higher age, higher frequencies of female sex, hypertension, diabetes mellitus, atrial fibrillation, and use of antithrombotic agents, and higher LVMI (Table 5). When the study population was grouped into baseline WMH volume quartiles and E/e’ intervals, the subgroup of E/e’ ≥15 in quartiles 1, 2, and 3 had significantly faster WMH progression rates compared to the E/e’ subgroups <15 (for quartiles 1, 1.92±1.86 mL/year vs. 0.10±0.27 mL/year, *P*<0.001; for quartiles 2, 1.87±2.04 mL/year vs. 0.28±0.61 mL/year, *P*<0.001; and for quartile 3, 2.32±1.00 mL/year vs. 1.12±1.30 mL/year, *P* = 0.009). However, in WMH volume quartile 4, the WMH progression rate among the E/e’ subgroups did not show a statistically significant difference (5.22±6.38 mL/year vs. 2.75±3.40 mL/year, *P* = 0.054, Fig 2).

**Table 5:** Comparison of clinical, echocardiography, and white matter hyperintensity profiles according to E/e’ ratio.

Clinical profiles	E/e’<8 (A, N = 147)	8≤ E/e'<15 (B, N = 185)	E/e’ ≥15 (C, N = 60)	*P*
Age, years	64.2±7.7	67.6±8.5	69.9±8.2	<0.001** A<B**, A<C**
Male sex	81 (55.1)	120 (64.9)	23 (38.3)	0.001** B>C**
Previous lacunar stroke	40 (27.2)	52 (28.1)	19 (31.7)	0.810
Hypertension	67 (45.6)	136 (73.5)	51 (85.0)	<0.001** A<B**, A<C**
Diabetes mellitus	34 (23.1)	68 (36.8)	32 (53.3)	<0.001** A<B*, A<C**
Hyperlipidemia	43 (29.3)	67 (36.2)	19 (31.7)	0.399
Subclinical coronary artery disease	9 (6.1)	40 (21.6)	8 (13.3)	<0.001** A<B**
Smoking in past 5 years	13 (8.8)	28 (15.1)	8 (13.3)	0.223
Atrial fibrillation	9 (6.1)	19 (10.3)	18 (30.0)	<0.001** B<C**, A<C**
Use of antithrombotic agents	96 (65.3)	149 (80.5)	54 (90.0)	<0.001** A<B**, A<C**
Use of beta-blockers	39 (26.5)	59 (31.9)	18 (30.0)	0.569
Use of ACEI/ARB	53 (36.1)	75 (40.5)	20 (33.3	0.526
**Echocardiography profiles**				
Ejection fraction, %	63.1±5.4	62.4±6.9	61.0±7.3	0.091
E/e’	6.9±1.1	10.8±1.6	17.2±2.7	<0.001**
Left ventricular mass index, g/m^2^	87.0±16.6	91.4±24.4	99.9±23.9	0.001**A<C**
**Radiography profiles**				
Baseline WMH volume, mL	2.42 (0.69–8.51)	5.25 (1.61–13.86)	5.61 (1.78–14.25)	0.008^†^** A<B**, A<C*
WMH progression rate, mL/year	0.08 (0.00–0.72)	0.49 (0.01–1.36)	1.87 (0.62–3.45)	<0.001^†^** B<C**, A<C**

Data are reported as number (percentage), as mean± standard deviation, or as median (interquartile range, IQR). ACEI/ARB: angiotensin-converting enzyme inhibitor/aldosterone-receptor blocker, WMH: white matter hyperintensity

^†^*P* values from Analysis of Covariance analysis adjusting for age and sex

*P<0.05, and

**P<0.01.

## Discussion

In the present study, we revealed that the echocardiography index E/e’ is associated with the WMH progression rate in populations with preserved systolic function. This association was significant after adjusting the previously recognized factors related with reduced arterial compliance, including age and conventional cerebrovascular risk factors. [1–3, 13, 14] As far as our knowledge, this might be the first to demonstrate the association between LV diastolic dysfunction and the long-term progression of cerebral WMH volume. Additionally, GFR and baseline WMH severity were also associated with the increased rate of WMH progression, which is in accordance with the previous reports. [12, 13]

As the heart primarily generates the pulsation of the cerebral small arteries, previous studies investigated the link between cardiac hemodynamics and the brain degenerative process. Reduced cardiac output, increased LVMI, and raised right atrial pressure have been shown to be associated with the development of dementia, compromised white-matter microstructure, and WMH volume, respectively. [27, 29, 30] However, E/e’ might have some advantages as it is a widely used echocardiographic marker with easy measurability that correlates with the long-term progression of cerebral WMH. [15, 17, 21, 22]

Notably, an elevated E/e’ (≥15) showed a faster WMH progression rate compared to E/e’ values of <15. The difference in the WMH progression rate among the E/e’ subgroups was more prominent in subpopulations with mild baseline WMH severities (WMH volume quartiles 1 and 2) than those with severe baseline WMH severities. Considering that age, frequencies of hypertension, diabetes mellitus, atrial fibrillation, and use of antithrombotic agents, and LVMI were also higher in the E/e’ ≥15 subgroup, an E/e’ value of ≥15 might indicate the activated pathologic processes of WMH progression although when the brain MRI appears grossly normal. Additionally, given that the cerebral WMH has a long-term progressive course, an E/e’ value of ≥15 might be used as a cut-off value for the increased risk of long-term WMH progression, especially for a geriatric population whose baseline WMH is minimal or mild.

The association of the E/e’ and the cerebral WMH progression may be supported by similarities in the mechanisms underlying the LV diastolic dysfunction and the reduced cerebral arteriolar compliance. Firstly, a common dysregulated remodeling process underlies both the LV diastolic dysfunction and the cerebral arteriolar stiffness, including chronic alteration of the amount, property, and cross-linking of the extracellular matrix, resulting in the increased passive tension in those systems. [3, 10, 20, 31–33] Secondly, chronic subclinical tissue inflammation in both systems induce increased vascular permeability and activation inflammatory cascades, resulting in fibrosis and degeneration of myocardium and brain parenchyma. [1, 5, 20, 34] Thirdly, sustained activation of the sympathetic and renin-angiotensin-aldosterone systems induces chronic elevation of the tone and endothelial dysfunction in both myocardial and cerebral arterioles, [1–20, 31] resulting in decreased reactivity to nitric oxide. [7]

One of the major limitations of the present study is that there are some issues for selection bias preventing the application of the findings of this study to the general population. First, although this study incorporated individuals without myocardial infarction or chronic central nervous system disorder, the study population was restricted to a single tertiary institution database with follow-up MRI evaluations. Since the population who developed major cardiovascular events or whose medical conditions had significantly deteriorated might have been prevented from performing follow-up MRIs, this might be a potential source of selection bias. Second, the mean value of LVMI was higher than normal values described in the population-based study (91.2±21.2 g/m2 vs. 69.9+17.5 g/m2), [35] which might be partially due to the high frequency of cardiovascular comorbidities in this study population. Methodological limitations of measuring E/e’ or WMH volume progression rate should also be addressed. First, we used septal e’ value rather than the average of septal and lateral e’ values to measure the E/e’. This might partially explain the high frequency of E/e’ elevation in the study and requires a careful interpretation of the study results. [22] Second, we did not co-registered the baseline and the follow-up images for measuring the WMH volume changes, although we co-registered and aligned the images on a template. [36] Third, although the magnetic field strength was harmonized, sequence harmonization between different scanners and matching of the MRI machines between the baseline and the follow-up were not performed, which might reduce the data reproducibility. Additionally, the interval between initial and follow-up MRI evaluations was not standardized due to the retrospective study design. Further prospective community-based studies with a standardized evaluation and follow-up protocol might resolve these issues.

## Supporting information

S1 Table Indications for the initial and follow-up evaluations. (DOCX)

S2 Table Comparison of the demographic, clinical, echocardiographic, and MRI parameters among the subgroups with different MRI scanners. (DOCX)

S1 Dataset Full dataset of the study population. (XLSX)
