# Supplementary material for: Echocardiographic index E/e’ in association with cerebral white matter hyperintensity progression
Source: PLoS One. 2020 Jul 27;15(7):e0236473. doi: 10.1371/journal.pone.0236473 (PMC7384642; doi:10.1371/journal.pone.0236473)
Supplement: S1 Table — (DOCX) [file pone.0236473.s001.docx]

**Supplemental S1 Table.** Indications for the initial and follow-up evaluations

| 1. Indications for initial MRI/MRA |  | Number (%) |
| --- | --- | --- |
| 1. Medical check-up program ^a^ | | 190 (48.5%) |
| 1. Evaluation for headache or dizziness ^b^ | | 103 (26.3%) |
| 1. Follow-up of an old lacunar infarction | | 99 (25.3%) |
| Indications for follow-up MRI/MRA | |  |
| 1. Medical check-up program | | 274 (69.9%) |
| 1. Follow-up of baseline WMH | | 16 (4.1%) |
| 1. Follow-up of an old lacunar infarction | | 102 (26.0%) |

MRI/MRA: magnetic resonance image/angiography, WMH: white matter hyperintensity. ^a^ Medical check-up program provided by Seoul National University Hospital Healthcare System for the aged population, ^b^ Primary or nonspecific headache in 78 patients and dizziness without a CNS origin in 25 patients.
