# Supplementary material for: Echocardiographic index E/e’ in association with cerebral white matter hyperintensity progression
Source: PLoS One. 2020 Jul 27;15(7):e0236473. doi: 10.1371/journal.pone.0236473 (PMC7384642; doi:10.1371/journal.pone.0236473)
Supplement: S2 Table — (DOCX) [file pone.0236473.s002.docx]

**Supplemental S2 Table.** Comparison of the clinical, e, and white matter hyperintensity profiles among the subgroups with different MRI scanners.

| **Clinical profiles** | Intera Achieva  (A, N=164) | Verio  (B, N=129) | Signa  (C, N=99) | *P* |
| --- | --- | --- | --- | --- |
| Age, years | 67.0±8.2 | 66.7±9.0 | 66.1±8.1 | 0.736 |
| Male sex | 86 (52.4) | 77 (59.7) | 61 (61.6) | 0.270 |
| Previous lacunar stroke | 47 (28.7) | 41 (31.8) | 23 (23.2) | 0.364 |
| Hypertension | 103 (62.8) | 85 (65.9) | 66 (66.7) | 0.778 |
| Diabetes mellitus | 52 (31.7) | 53 (41.1) | 29 (29.3) | 0.121 |
| Hyperlipidemia | 57 (34.8) | 41 (31.8) | 31 (31.3) | 0.803 |
| Subclinical coronary artery disease | 25 (15.2) | 14 (10.9) | 18 (18.2) | 0.283 |
| Smoking in past 5 years | 16 (9.8) | 22 (17.1) | 11 (11.1) | 0.154 |
| Atrial fibrillation | 13 (7.9) | 19 (14.7) | 14 (14.1) | 0.138 |
| Use of antithrombotic agents | 127 (77.4) | 95 (73.6) | 77 (77.8) | 0.692 |
| Use of beta-blockers | 51 (31.1) | 33 (25.6) | 32 (32.3) | 0.468 |
| Use of ACEI/ARB | 48 (29.3) | 56 (43.4) | 44 (44.4) | 0.013^*^  A<B^*^, A<C^*^ |
| **Laboratory findings** |  |  |  |  |
| Glomerular filtration rate, mL/min | 73.6±22.5 | 73.4±22.9 | 75.2±20.6 | 0.805 |
| Systolic BP, mmHg | 135.1±23.1 | 132.7±22.2 | 134.3±21.6 | 0.650 |
| Pulse pressure, mmHg | 56.4±17.5 | 55.3±16.2 | 54.5±14.7 | 0.659 |
| **Echocardiography profiles** |  |  |  |  |
| Ejection fraction, % | 62.3±6.4 | 63.1±6.3 | 61.9±6.6 | 0.384 |
| E/e’ | 10.4±3.9 | 9.9±3.5 | 10.7±4.0 | 0.360 |
| Left ventricular mass index, g/m^2^ | 92.1±23.9 | 92.8±20.7 | 87.1±20.3 | 0.111 |
| **Radiography profiles** |  |  |  |  |
| Baseline WMH volume, mL | 3.61 [1.27–12.37] | 4.24 [1.32–12.21] | 3.54 [1.17–15.27] | 0.188 |
| WMH progression rate, mL/year | 0.5 [0.01–1.41] | 0.22 [0–1.32] | 0.44 [0.01–1.73] | 0.171 |

Data are reported as number (percentage), as mean± standard deviation, or as median (interquartile range, IQR). BP: blood pressure, ACEI/ARB: angiotensin-converting enzyme inhibitor/aldosterone-receptor blocker, WMH: white matter hyperintensity, ^*^*P*<0.05.
